# Irrigation affects characteristics of narrow-leaved lupin (*Lupinus angustifolius* L.) seeds

**DOI:** 10.1007/s00425-019-03091-9

**Published:** 2019-01-25

**Authors:** Konrad Krajewski, Iwona Ciereszko, Joanna Leśniewska, Alina T. Dubis, Anna Basa, Aneta Żabka, Marcin Hołota, Łukasz Sobiech, Agnieszka Faligowska, Grzegorz Skrzypczak, Janusz Maszewski, Justyna T. Polit

**Affiliations:** 1 Department of Cytophysiology, Faculty of Biology and Environmental Protection, University of Łódź, ul. Pomorska 141/143, 90-236 Lodz, Poland; 2 Faculty of Biology and Chemistry, Institute of Biology, University of Bialystok, Ciołkowskiego1J, 15-245 Bialystok, Poland; 3 Faculty of Biology and Chemistry, Institute of Chemistry, University of Bialystok, Ciołkowskiego1K, 15-245 Bialystok, Poland; 4 Agronomy Department, Poznań University of Life Sciences, Dojazd 11, 60-632 Poznan, Poland

**Keywords:** Endoreplication, FTIR, Germination, Mitotic activity, SEM–EDX, Storage proteins, Stress memory

## Abstract

While plant irrigation usually increases yield, irrigation also affects seed characteristics with respect to endoreplication level, chemical composition, number of carbonyl bands, and cuticular wax profiles.

Seeds of sweet varieties of the narrow-leaved lupin have good nutritional properties; however, these plants are sensitive to water deficit. Irrigation improves lupin yield, but can affect seed characteristics. The purpose of the study was to evaluate irrigation influence on lupin seed features and their chemical composition. Morphological analyses showed worse quality of seeds from the irrigated plants, with regard to their size and weight. This was confirmed by cytophotometric analyses which revealed a lower DNA content in the nuclei of cells from the apical and basal regions of the irrigated seeds. The lower degree of polyploidy of the nuclei entails lower cell sizes and limited space for storage components. Fourier transform infrared spectroscopic analysis demonstrated that protein and cuticular wax profiles of the irrigated seeds were different from the control. The electrophoretic analyses indicated differences in protein profiles including changes in the proportion of lupin storage proteins. Among the various studied elements, only the nitrogen content decreased in the embryo axis of irrigated plants. Although germination dynamics of the irrigated seeds was higher, the seedlings’ development rate was slightly lower than in the control. The hydrogen peroxide level in root meristem cells was higher during germination in the control suggesting its regulatory role in seed metabolism/signaling. Our study indicated that irrigation of lupin plant affected seed features and composition.

**Electronic supplementary material:**

The online version of this article (10.1007/s00425-019-03091-9) contains supplementary material, which is available to authorized users.

## Introduction

Global climate change is a source of unusual weather phenomena which include drought and floods. Water shortage induces activation of plant stress responses which affect plant growth and development. Reduced chlorophyll content, low CO_2_ uptake, and reactive oxygen species (ROS)-mediated damages of the photosynthetic apparatus decrease the rate of photosynthesis. All changes in the plant metabolism which ensure survival of unfavorable conditions limit crop yield, including that of sensitive to water-deficit legumes (Gresta et al. 2017). Every unexpected and extended reduction of agricultural productivity causes economic losses among farmers and increases food prices. In an extreme situation, prolonged drought correlated with world overpopulation could cause hunger and social tensions (Almer et al. 2016). Thus, improvement of crop tolerance to drought is the main concern of agriculture-related studies, nowadays (Cantale et al. 2018).

Legume plants, including lupin species, are of great importance both in terms of nutrition, agriculture, and economics. Narrow-leaved lupin, yellow lupin, and white lupin intended for seeds, green fertilizer, and for feed are predominantly cultivated. As rotation crops, they play a phytosanitary role, improve soil structure, and are able to fix nitrogen. As post-harvest residues, they enrich the soil in macro- and micronutrients, increase the yield of follow-up plants, and break the aftermath of cereals. However, their yielding is unstable under unfavorable weather conditions (e.g., drought) during vegetation. Water shortage leads to falling of flowers, smaller number of pods, and low yield. In addition, water-deficit conditions can influence seed chemical composition, e.g., increasing alkaloid content in some sweet lupin varieties (Christiansen et al. 1997; Gresta et al. 2017; Hane et al. 2017).

During plant growth and development, also under stress conditions, endoreduplication is found to play an important role and is considered being relevant for plant fitness (Scholes and Paige 2015). Nuclear DNA content is related to cell size and endoreduplication induces cell expansion and fast growth. This mechanism seems to be advantageous, especially when energy is limited or rapid growth is necessary. Terminal differentiation of some cells and their specialized functions also depend on endocycles (Lee et al. 2009). Endoreduplication may be associated with the production of storage metabolites in cereal endosperm and legume cotyledons (Knake-Sobkowicz and Marciniak 2005; Lee et al. 2009; Dante et al. 2014). However, the correlation between endoreduplication and accumulation of storage proteins was contradicted in some studies (Leiva-Neto et al. 2004). Exogenous stresses may also induce endoreduplication in some tissues to maintain their function and to increase plant fitness fortifying them against adverse environment. Thus, changes in the genome size may reflect the response to environmental stress and polyploidy seems to be a conserved mechanism developed during evolution, allowing for survival under unfavorable conditions (Cookson et al. 2006; Lee et al. 2009; Madlung 2013; Scholes and Paige 2015).

Drought is one of the most devastating abiotic stresses; however, plants developed different strategies to cope with water shortage including drought escape, drought avoidance, or drought tolerance strategies. Water stress avoidance allows maintaining high water potential in cells due to decreased leaf areas and expanded root systems. Drought tolerance is an ability to endure low water potential by, e.g., accumulation of osmolytes increasing the osmotic potential and higher water uptake by roots. Shortage of water is accompanied by oxidative stress and excessive formation of ROS may cause damages in photosystems or cell membranes (Moran et al. 1994; Farooq et al. 2009; Osakabe et al. 2014; Yadav and Sharma 2016).

Since shortage of water reduces crop yield, intensive research to produce drought resistant crops is being conducted. However, the complexity of responses to drought makes these studies difficult and time-consuming (Farooq et al. 2009; Yadav and Sharma 2016; Cantale et al. 2018). Thus, different methods of irrigation are still the most common approach to reduce adverse effects of water deficit in agriculture. It was found to increase crop productivity, including legumes (Gresta et al. 2017), and it may have positive effects on seeds quality (Breen and Richards 2008). However, there are contradictory results, indicating that irrigation might reduce seed vigor, germination energy, and germination capacity in lupin (Faligowska et al. 2016). Thus, one may ask whether irrigation is advantageous in all circumstances and whether it should be always applied when plants are exposed to mild water stress only. If seeds are used as feed for animals, or for consumption purposes, the high yield is desirable. However, if seeds are treated as planting material, the answer is not so obvious. We have to take into consideration the fact that although mild drought reduces crop productivity, it may increase plant resistance to stress with naturally occurring mechanisms (Backhaus et al. 2014). Stress memory would help to deal with unfavorable conditions in the next plant generation (Walter et al. 2011; Crisp et al. 2016; Fleta-Soriano and Munné-Bosch 2016; Li and Liu 2016).

The aim of this study was to investigate changes at cytological, chemical, and biochemical levels which may be responsible for features of seeds obtained from irrigated plants of *Lupinus angustifolius*. The presented study indicates that seedlings obtained from the irrigated and non-irrigated plants differ in mitotic activity, ROS level, endoreplication level, and protein profile estimated during the initial days of germination. Differences in the number of carbonyl bands and in cuticular wax profiles of epidermal cells as well as chemical elements were also found between seeds from the irrigated and non-irrigated plants.

## Materials and methods

### Plant cultivation

The research was conducted on the basis of a field experiment in three consecutive years carried out at the Złotniki Research Station (52°29′N, 16°49′E, Złotniki, Poland), Poznań University of Life Sciences. The experiment was laid out in a randomized complete block design with 4 replications. The study was conducted as a stationary experiment on grey–brown podzolic soil (pH 4.8 measured in 1 M KCl; 1.3% organic matter: 50–110 mg kg^−1^ P, 115–195 mg kg^−1^ K) in 4-crop rotation. A narrow-leaved lupin, *Lupinus angustifolius* cultivar Baron (from plantation HR Smolice, Poland, at a rate of 150 kg ha^−1^), was sown in early April. Sowing depth was 4 cm and the row distance was 18 cm. The main plot treatments were natural rainfall (non-irrigated) and natural rainfall plus irrigations (irrigated). There was a gap of 6 m in width between non-irrigated and irrigated parts of plots. Irrigations were applied when consumption of 30% of the readily available soil moisture was observed in the 0.30 m root zone during flowering, pod, and seed ripening (May, June, and July). The soil moisture content was measured by the gravimetric method. Irrigation water was taken from a small reservoir near the experimental site. The quality of irrigation water used in the study was good. Irrigation was performed using a water pump with outlet pipes and a rotary sprinkler. Aluminium pipes of 110 mm in diameter were used. The diameters of the nozzles were 20 mm and the discharge rate was 5 l h^−1^. The main pipes with the rotary sprinkler were placed in the middle of irrigated parts of plots. The mean dose of water and time of irrigation during vegetation period were 30–35 mm and 6-7 h, respectively (Suppl. Table S1), while the mean daily air temperatures and total precipitation in the vegetation periods in May, June, and July were 15.3, 18.4, and 17.5 °C and 17.5, 62.4, and 214.8 mm, respectively (Suppl. Table S2), data from the Agrometeorological Observatory in Złotniki. Therefore, the irrigated plants received approximately 49% of water more compared to the control plants.

### Yield assessment

Ten whole plants of narrow-leaved lupin were collected randomly 2 days before harvest and were used to measure seed yield (expressed as g per plant).

### Seed germination and culture for cytological research

For cytological research, seeds of lupin were sown in Petri dishes on filter paper soaked with distilled water (10 seeds/∅ 15 cm) and germinated in darkness at room temperature for maximum 4 days.

### Feulgen-staining and cytophotometry

Cotyledons and apical fragments of embryo roots were fixed in cold Carnoy’s mixture of absolute ethanol and glacial acetic acid (3:1; v/v) for 1 h. Following fixation, the roots were washed several times with ethanol, rehydrated (70–30% ethanol, distilled water), hydrolyzed in 4 M HCl for 1 h, and stained with Schiff’s reagent (pararosaniline; Sigma-Aldrich, Poznań, Poland). After rinsing in SO_2-_water (three times) and distilled water, fragments of cotyledons from the selected zones and apical segments of the roots were cutoff, placed in a drop of 45% acetic acid, and squashed onto Super-Frost (Menzel-Gläser, Braunschweig, Germany) microscope slides. Following freezing with dry ice, cover slips were removed, and the dehydrated dry slides were embedded in Canada balsam. Nuclear DNA content was evaluated by means of microdensitometry using a Jenamed 2 microscope (Carl Zeiss, Jena, Germany) with the computer-aided Cytophotometer v1.2 (Forel, Lodz, Poland). The Feulgen-stained cell nuclei were measured at 565 nm. The preparates made using this method were used also to analyze the mitotic and phase indexes.

### Electrophoretic separation of proteins

P-PER Plant Protein Extraction Kit (Thermo Fisher Scientific, Rockford, IL, USA) supplemented with Protease Inhibitor Cocktail was used for total protein extraction. The Lowry et al. (1951) procedure was used to determine the total level of proteins in the solution. Whole-cell protein extracts were fractionated on NuPAGE^®^ Novex^®^ 4–12% Bis–Tris gel, in NuPAGE^®^-Mes SDS (50 mM Mes, 50 mM Tris, 0.1% SDS, 1 mM EDTA) buffer (pH 7.3; 200 V; 110-125 mA; Thermo Fisher Scientific). Analysis of staining intensity (Coomassie™) of the bands obtained by the electrophoretic separation of proteins was carried out using the Gel Analyzer 2010a.

### FTIR analysis of lupin seeds

Infrared spectroscopy is an analytical technique offering a possibility of chemical identification of samples. This FTIR technique is based on the fact that chemical substances show selective absorption in infrared regions. The molecules vibrate, after absorption of IR radiations, giving rise to the spectrum of absorption (Dubis et al. 2001). The FTIR spectra were recorded in the range between 4000 and 500 cm^−1^ with a Thermo Scientific Nicolet™ 6700 spectrometer. The spectra were recorded at a resolution of 4 cm^−1^, apodized with triangular function, and zero-filling factor of 1 was applied. DRIFTS spectra were recorded using a Spectra-Tech diffuse reflectance accessory equipped with the Si-Carb Sampling Kit (Spectra-Tech Inc, Hanover Park, IL, USA). The sample was analyzed directly on the sample cup after roughing it with abrasive paper. A small disc of silicon carbide paper was used to abrade the sample to be analyzed. Pieces of clean silicon carbide paper were used as the background. The sample spectrum was rationed against a background spectrum. For the Fourier transform infrared spectroscopy/horizontal attenuated total reflectance technique (FTIR/HATR), a diamond crystal was used. HATR technique provides a simple means of direct handling of plant material. The lupin samples were placed in HATR cells and a beam of infrared radiation entering a crystal underwent single internal reflections. The resultant radiation was measured and plotted as a function of the wave number.

### Scanning electron microscope/energy-dispersive X-ray spectroscopy (SEM/EDS) microanalysis

SEM was used for morphological analysis of seed samples, and the EDS technique to identify different chemical elements present in lupin seeds as described by (Psaras and Manetas 2001), with modifications. Mature, dry seeds of lupin from control and irrigated plants (5 seeds of each kind), were cut on half and were observed without sputter coating with gold with an SEM, model FEI INSPECT S50. X-ray microanalyses were made with the EDAX system (Ametek, Weiterstadt, Germany) connected to the SEM, in six selected points of each seed (embryo axis, cotyledon near plumule, cotyledon center, cotyledon near radicle, seed coat, and plumule, Fig. 5a, b). In all cases, the voltage was 20 kV (for micrographs 10 kV), the pressure 60 Pa, spod size 3, and live time 30 s. EDS spectra were analyzed and elements, whose presence was recorded in the form of peaks summarized in tables (eZAF Smart Quant Results, EDAX). The content of chemical elements (weight %) was estimated statistically.

### Histochemical localization of H_2_O_2_

The generation of H_2_O_2_ was observed using peroxidase-catalyzed 3,3-diaminobenzidine (DAB; Sigma-Aldrich) polymerization test. The experimental procedure was performed according to Thordal-Christensen et al. (1997) with some modifications (Żabka et al. 2012). Three-day-old seedlings of lupin were incubated in a solution containing 1 mg mL^−1^ DAB dissolved in Tris buffer (10 mM Tris, 10 mM EDTA-2Na, 100 mM NaCl, pH 7.6) for 12 h. Additional control series comprised lupin seedlings incubated with 1 mM ascorbic acid (AA; Sigma-Aldrich). Then, roots were fixed for 40 min (4 °C) in PBS-buffered 3.7% paraformaldehyde solution, washed three times with PBS, and placed in a citric acid buffered digestion solution (pH 5.0) containing 2.5% pectinase, 2.5% cellulose, and 2.5% pectolyase, and incubated at 37 °C for 30 min. After that, the roots were washed with PBS, rinsed with distilled water, and squashed onto microscope glass slides in a mixture of glycerol and PBS (9:1; v/v). H_2_O_2_ was visualized under the microscope (SMZ-2T—equipped with DXM 1200 CCD camera Nikon) as a reddish-brown coloration.

### Statistical analysis

The differences between values obtained in the particular experiments were assessed with the analysis of variance (Anova) and following post hoc Tukey’s test or LSD test, the Student’s *t* test, or the Mann–Whitney *U* test. The choice of the test to the individual experiment was indicated in the description of the graphs.

## Results

### Seed yield

Irrigation significantly increased seed yield of the narrow-leaved lupin which was even 2.5 times higher than in non-irrigated plants (Suppl. Table S3). The seeds collected from main stem were of the best quality (in terms of visual appearance), while those from branches which maturated later were smaller. Thus, the site of seeds origin had huge importance for their size.

For clarity, in “Results and Discussion”, the seeds collected from the plants growing under natural conditions (without additional irrigation) as well as the seeds from the plants that were subjected to irrigation are referred to as “control seeds” and “irrigated seeds”, respectively.

### Seed morphology

**Fig. 1:**
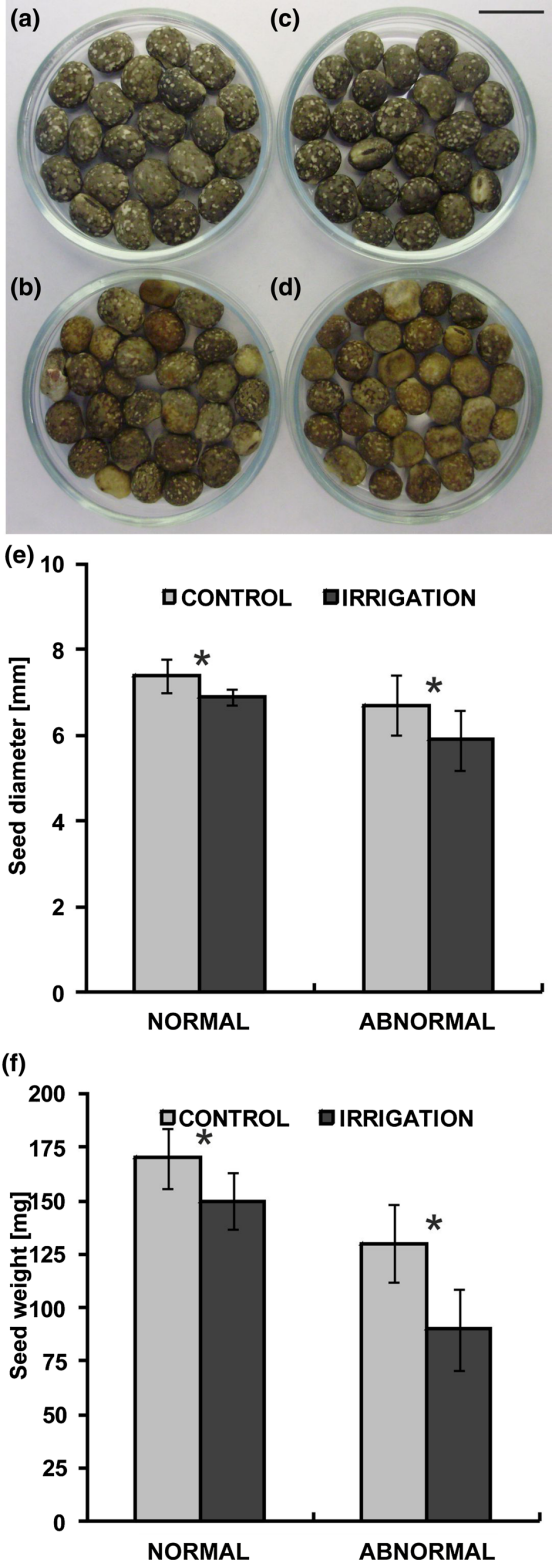
Seeds of narrow-leaved lupin collected from the control (not irrigated) and irrigated plants and sorted according to morphological features. **a** Control normal. **b** Control abnormal. **c** Irrigated normal. **d** Irrigated abnormal. Scale bar 10 mm. **e** Seed size (diameter) measured along the long axis. **f** Seed weight. Statistical significance between mean values of seed diameters and seed weights was assessed with the Mann–Whitney *U* test (*P* < 0.01) and Student’s *t* test (*P* < 0.01), respectively. Error bars represent standard deviation (SD). Asterisk indicates statistical significance between control and irrigated plants

The control seeds as well as the irrigated seeds were divided into normal and abnormal due to differences in their morphological characteristics (Fig. 1a–d). The former were large in size, kidney-shaped, slightly flattened and covered with a specific grey seed coat with a regular marble pattern. The latter were extremely small, with distorted (often concave) oval shape, brownish, or slightly stained, without the clear marble pattern. The size and weight of the seeds in each group indicated the inferior quality of the yield from the irrigated plants (they were smaller and lighter, Fig. 1e and f).

Because the percentage of abnormal seeds in both groups of plants was similar, i.e., 35% and 38% for the control and irrigated plants, respectively, as well as more than 60% of the abnormal ones did not germinate, in subsequent studies, only normal seeds were taken into account.

### DNA content

**Fig. 2:**
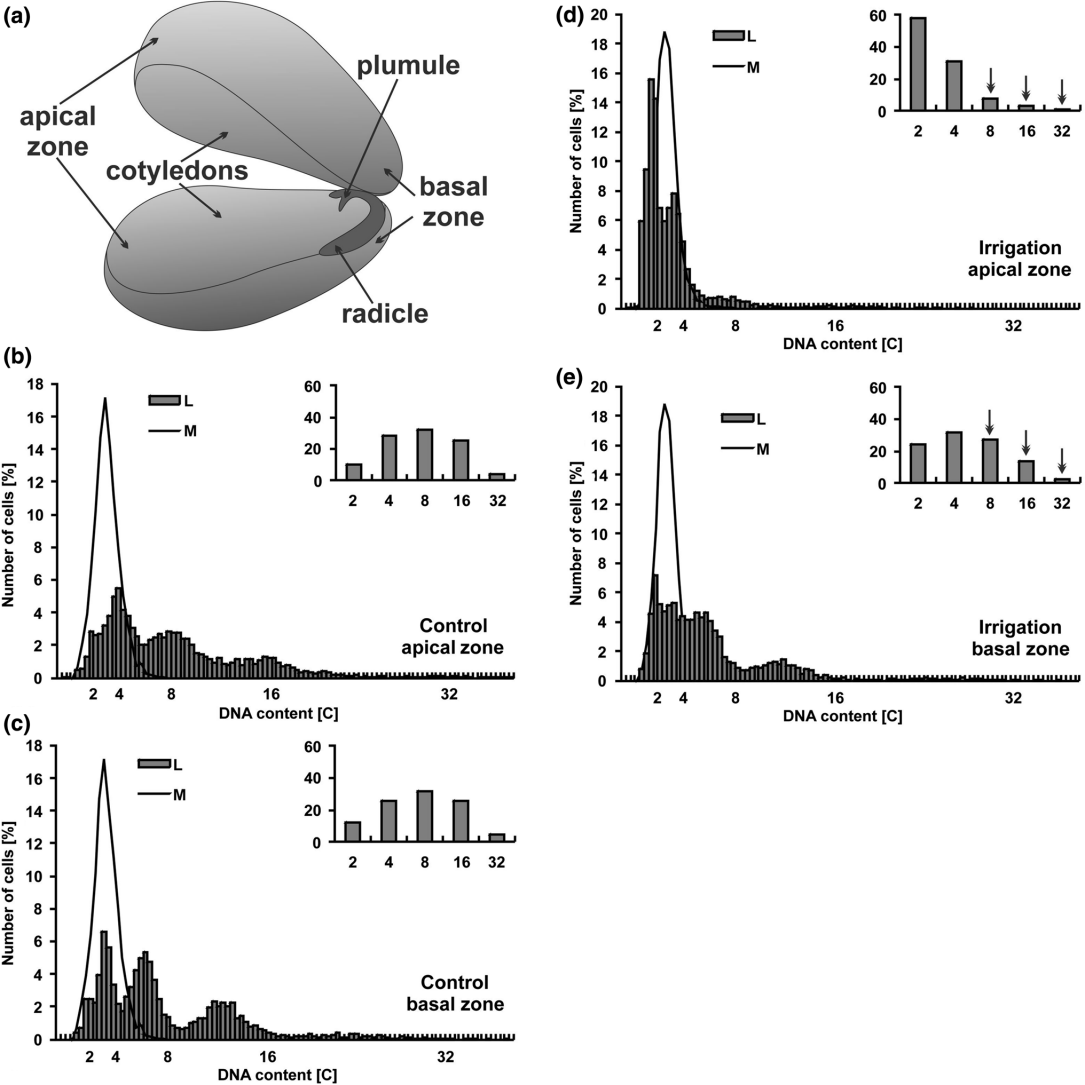
DNA content in the indicated zones of narrow-leaved lupin seeds. **a** Structure of lupin seed. **b**–**e** Frequency distribution [%] of nuclear DNA content in the selected zones: cotyledon zones (L) or root meristems (M) of lupin. **b** Apical zone of control seeds from not irrigated plants. **c** Basal zone of control seeds from not irrigated plants. **d** Apical zone of seeds from irrigated plants. **e** Basal zone of seeds from irrigated plants. Inserted bar graphs show percentages of cells after successive rounds of endoreplication. Arrows show decrease in the number of polyploid cells in the irrigated seeds

Cotyledons are the largest parts of lupin seeds. Cells in particular cotyledon zones may contain nuclei of different ploidy levels and thus be of different sizes. Cytophotometric measurements of nuclear DNA content in two extremely situated cotyledon zones (apical and basal, Fig. 2a) did not reveal differences in DNA content in the control seeds (Fig. 2b, c). In both zones, beside the 2C and 4C DNA cells (nearly 40%), mostly polyploid ones were observed (60%). More than 30% of them passed the first round of endoreplication and contained 8C DNA, while 25% passed two rounds of endoreplication reaching 16C DNA. A few (about 4%) contained 32C DNA.

However, considerable differences were seen when the ploidy level was compared between control and irrigated seeds. In the case of irrigated seeds, a significantly lower number of polyploid cells were observed in both zones, and a more pronounced difference was seen in the apical zone (Fig. 2d, e). In this case, cells with 2C and 4C DNA content characteristic of G1 and G2 phases of the cell cycle accounted for nearly 90% and polyploid only for 11% which were mainly those that reached 8C DNA (Fig. 2d). In the basal zone, the cells containing both 2C and 4C DNA represented more than half of the population (56%) and the polyploidy cells only 44%. Decrease in the number of polyploid nuclei in this zone was mainly related to a significant reduction of the cells with the second and third rounds of endoreplication (Fig. 2e).

### Protein profile

**Fig. 3:**
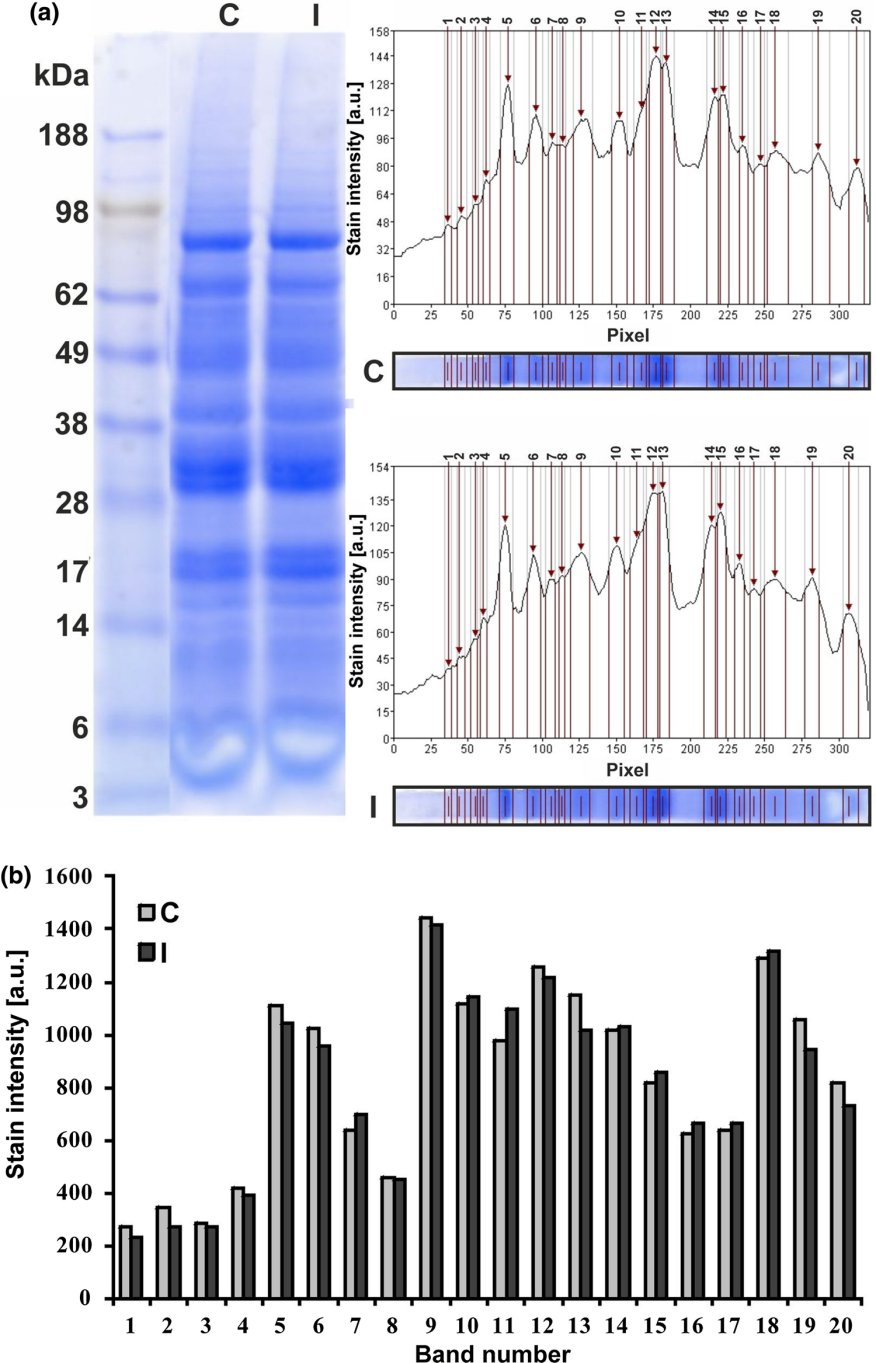
Protein profile in lupin cotyledons coming from the seeds collected from not irrigated (control C) and irrigated (I) plants. **a** Electrophoretic separation of proteins in polyacrylamide gel (stained with Coomassie Blue) and computer analysis of staining intensity of the detected bands. The first channel contains protein mass standard, the second and the third channel contain proteins coming from not irrigated (control C) or irrigated (I) seeds, respectively. **b** Comparison of protein contents in 20 detected bands

Analysis of the protein profile revealed by their electrophoretic distribution in the polyacrylamide gel allowed the assessment of differences in protein composition between the seeds from the control and the irrigated plants (Fig. 3a, b). The same number of distinguishable bands in both electrophoretic channels indicated the presence of a similar protein composition in the tested seeds, while the computer analysis of the intensity of their staining pointed to some differences in the amount of proteins present in them. The most stained bands (5, 6, 10, 12, 13, 14, and 15) contained subunits of storage proteins [globulins: γ-conglutin, β-conglutin (vicilin-like), and α-conglutin (legumin-like)] which are the most abundant in storage tissues of lupin. The decrease in storage protein content in some bands (5, 6, 12, and 13) and the increase in others (11, 14, and 15) indicated modifications of their proportion in the seeds of irrigated plants.

### FTIR analysis of lupin seeds

**Fig. 4:**
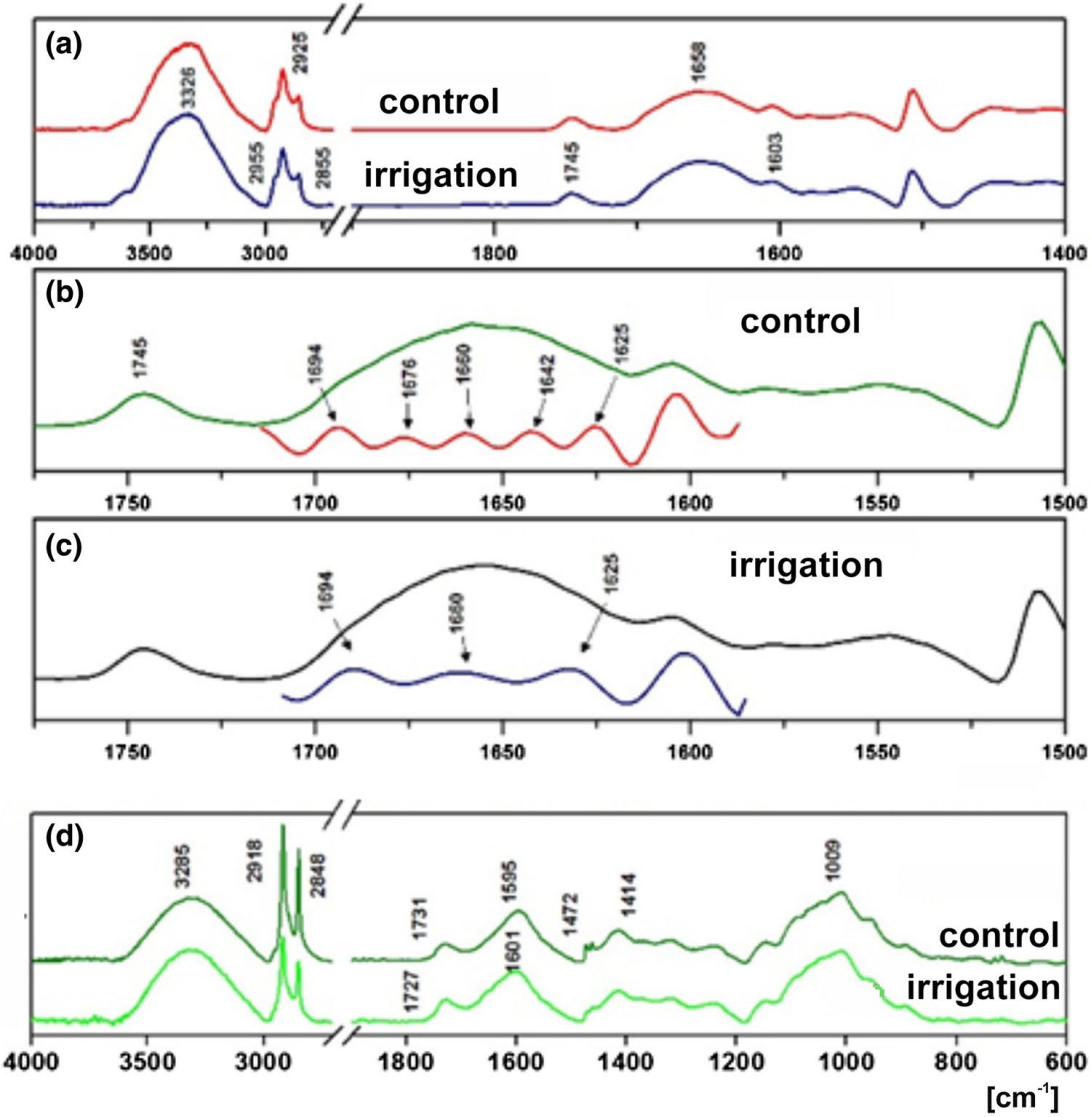
FTIR spectrum of the lupin seeds. **a** Peeled seeds collected from the control (not irrigated) and irrigated lupin plants. **b** Separation of the overlapping bands in the spectrum of the control seed, five Gaussian lines (red line) were found at 1694, 1676, 1660, 1642 and 1625 cm^−1^. **c** Separation of the overlapping bands in the spectrum of the irrigated seed, three Gaussian lines (navy line) were found at 1694, 1660, and 1625 cm^−1^. Spectra were recorded at room temperature using DRIFTS module. **d** ATR/FTIR spectrum of the lupin seed coat cutine from the control and irrigated material. Spectra were recorded at room temperature using ATR module

Figure 4 shows the FTIR spectra of a peeled lupin seed. In the spectrum of the control lupin seed (Fig. 4a), there are two prominent absorption bands at 3326 and 1658 cm^−1^ which could be assigned to N–H- and C=O-stretching bands. There are also three prominent bands at 2955, 2925, and 2855 cm^−1^ which could be identified as belonging to the aliphatic CH_3_ and CH_2_ groups. The signal at 1745 cm^−1^ may be treated as C=O-stretching vibration of triglyceride ester group. In addition, carbonyl absorption in the range of 1700–1620 cm^−1^ is present. Overlapping bands may be separated using the Fourier self-deconvolution method—FSD (Tooke 1988). The spectra of proteins exhibit absorption bands associated with their characteristic amide groups. The exact wave numbers of C=O vibrations depend on the nature of hydrogen-bonding interaction involving C=O and N–H groups. As a consequence, the amide I bands consist of a number of overlapping component bands. Thus, the FSD-IR was used to extract individual components from a complex composite band of C=O groups. Using this method, the *ν*_C=O_-stretching bands at 1694, 1676, 1660, 1642, and 1625 cm^−1^ were identified (Fig. 4b). Changes in the composition of the seed storage proteins due to the irrigating process (Fig. 4a, navy line) were observed. There were no absorption bands at 1676 and 1642 cm^−1^ (Fig. 4c, navy line) as compared with the spectrum of the control lupin seeds (Fig. 4b, red line). The difference of wave number reflects the structural nonequivalence of carbonyl groups. It means that various protein structures are present in the lupin seed.

Figure 4d shows ATR/FTIR spectra of lupin seed coats. There are four main absorption bands. The broad and intense band at 3285 cm^−1^ was assigned to the O–H-stretching modes of alcohols and fatty acids. The bands in the region of 2918–2848 cm^−1^ were assigned to the stretching of aliphatic CH_2_ groups. The band at 1732 was assigned to the C=O mode of ester group, while the broad band centered at 1595 cm^−1^ suggested the presence of proteins and that at 1009 cm^−1^ cellulose (Ribeiro da Luz 2006). Figure 4d illustrates ATR spectral differences between lupin seed cutine of the control and irrigated plants. The absorption band corresponding to the stretching of aliphatic CH_2_ groups was more intense for the control than for the irrigated seeds. In the carbonyl stretching region, the bands of strong intensity at 1595 cm^−1^ for non-irrigated and at 1601 cm^−1^ for irrigated seeds were observed.

### Analysis of chemical elements by SEM/EDS technique

**Fig. 5:**
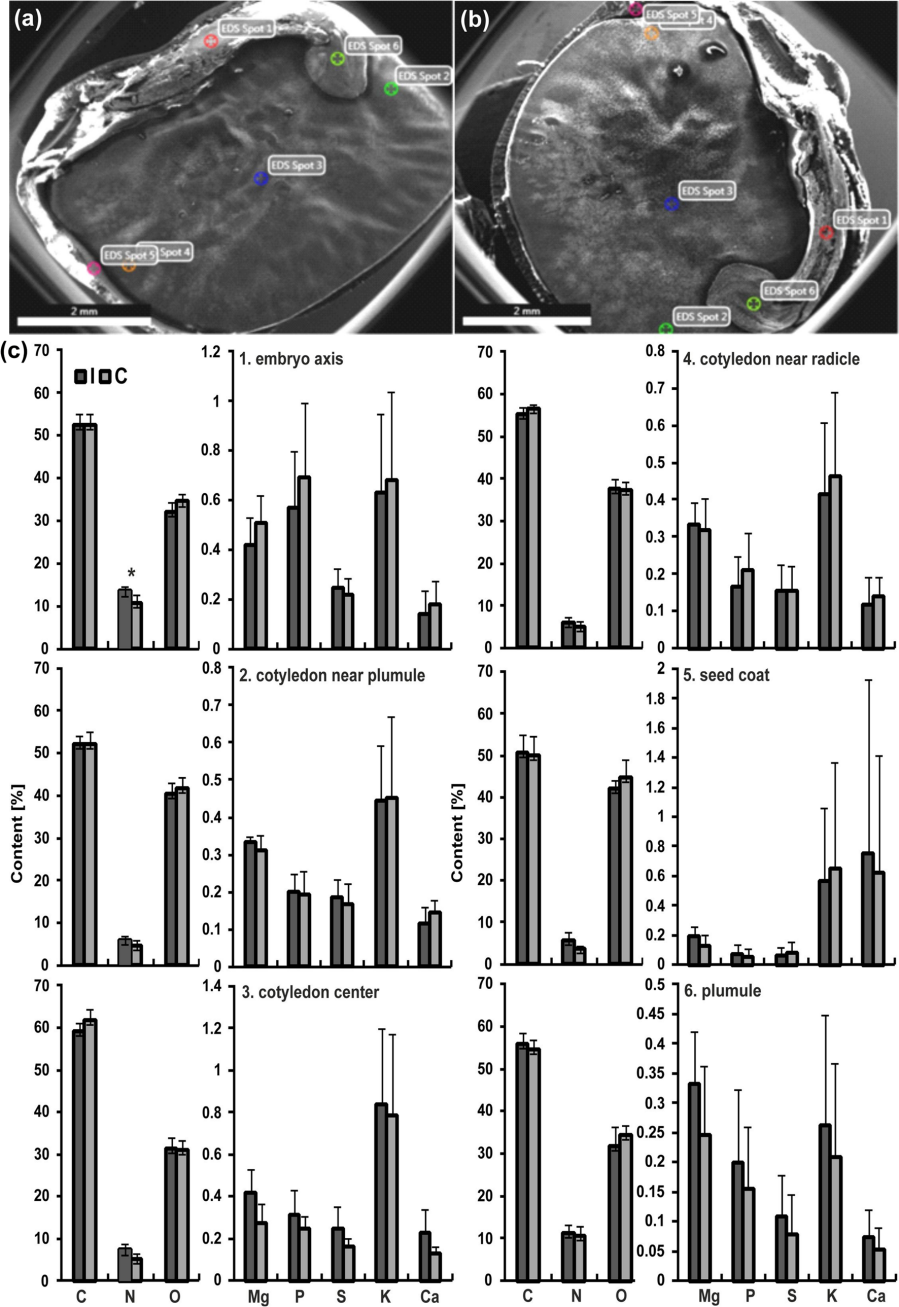
Scanning electron microscope (SEM) micrographs of lupin half seeds. **a** Seed of a control plant. **b** Seed of an irrigated plant. The points of elemental analysis in seeds are marked. Spots: 1, embryo axis; 2, cotyledon near plumule; 3 cotyledon center; 4 cotyledon near radicle; 5, seed coat; 6, plumule. **c** Corresponding content (weight %) of chemical elements (C, N, O, Mg, P, S, K, and Ca) in the indicated zones of the seeds (C, control; I, irrigated plants, respectively). Statistical significance between mean values was assessed with the Student’s *t* test (*P *= 0.03). Error bars represent standard deviation (SD). An asterisk indicates statistically significant results

Three chemical elements, C, O, and N, were dominant in the analyzed narrow-leaved lupin seed zones—their contents were similar in the control and irrigated seeds (Fig. 5). Among the various studied elements, only the nitrogen content decreased (statistically significant difference) in the embryo axis as a result of plant irrigation. Other elements (Mg, P, S, K, and Ca), whose presence was recorded in the form of peaks and whose contents (expressed in weight %) oscillated around 1% showed no statistically significant changes after irrigation treatments (Fig. 5c).

### Germination and seedlings’ growth

**Fig. 6:**
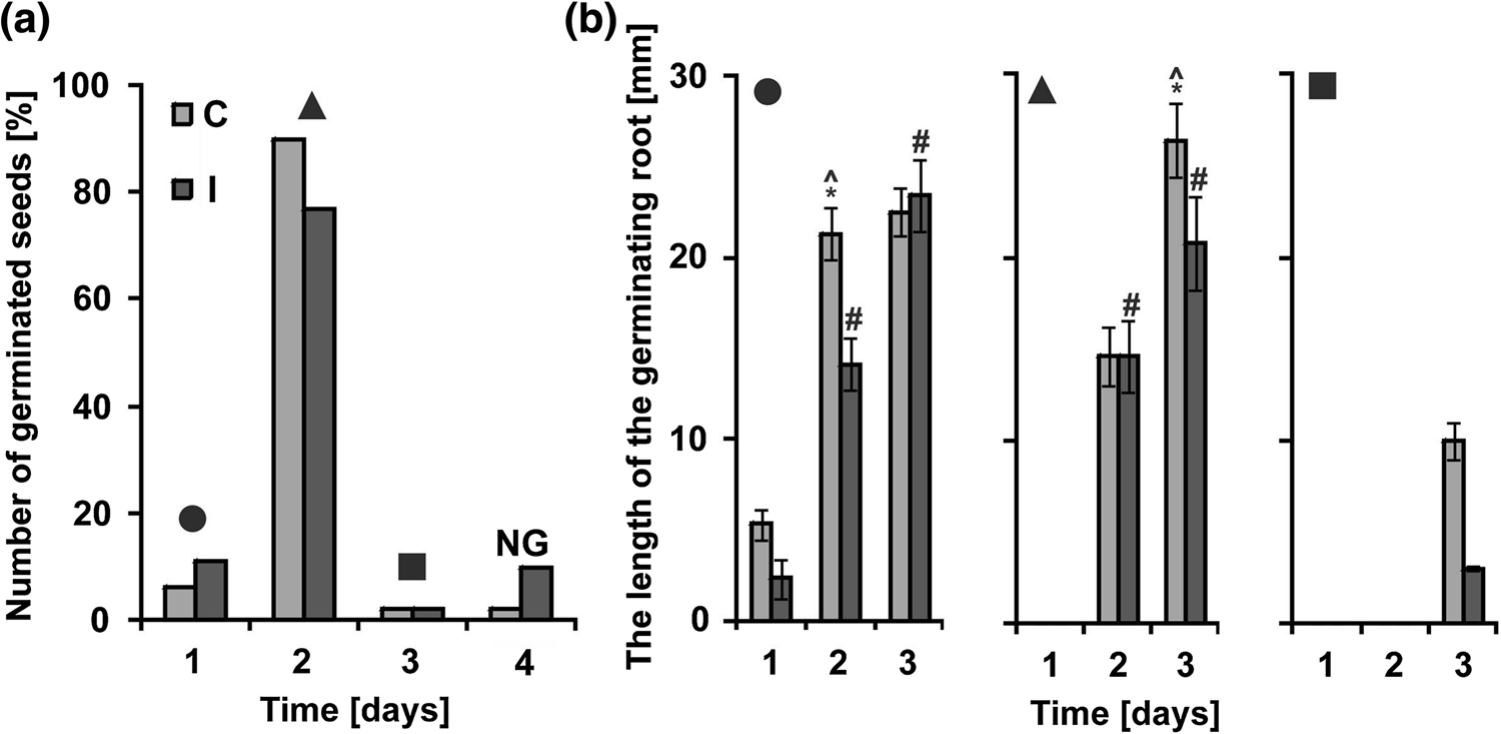
Dynamics of lupin seed germination and growth of embryo roots. **a** Percentage of germinated seeds during 3 days. The seeds collected from not irrigated plants (control C). The seeds collected from irrigated plants (I). The seeds which remained non-germinated after 4 days (NG). Black figures (circle, triangle, and square) above bars indicate populations of germinated seeds, whose root length is presented on the graphs marked with an adequate figure in part **b**. **b** Dynamics of embryo roots growth during following days of germination. Statistical significance between mean values in diagram marked with black circle and triangle was assessed with the two-way Anova with the post hoc unequal N HSD Tukey test (*P *< 0.01) and the Mann–Whitney *U* test (*P* < 0.01), respectively. Error bars represent standard deviation (SD). Asterisk indicates statistical significance between seedlings from control and irrigated seeds, and circumflex and hash symbols indicate statistical significance between seedlings from control or irrigated seeds, respectively, during following days of growth

Substances stored in the cotyledons are used during germination and growth of young seedlings. The dynamic of these processes is the parameter that allows evaluating the seed quality for sowing. The seeds collected from control and irrigated plants were germinated for 3 days (Fig. 6). After the first day of germination as much as 11% of the irrigated seeds sprouted out, while in the control only 6% (Fig. 6a). In both groups of seeds, the majority sprouted after 2 days. However, the percentage of germinated normal seeds was higher in the control (98%) than those in the irrigated material (90%). The seedlings that sprouted from the seeds of irrigated plants grew at a much slower rate than the control ones (Fig. 6b).

### Mitotic activity

**Fig. 7:**
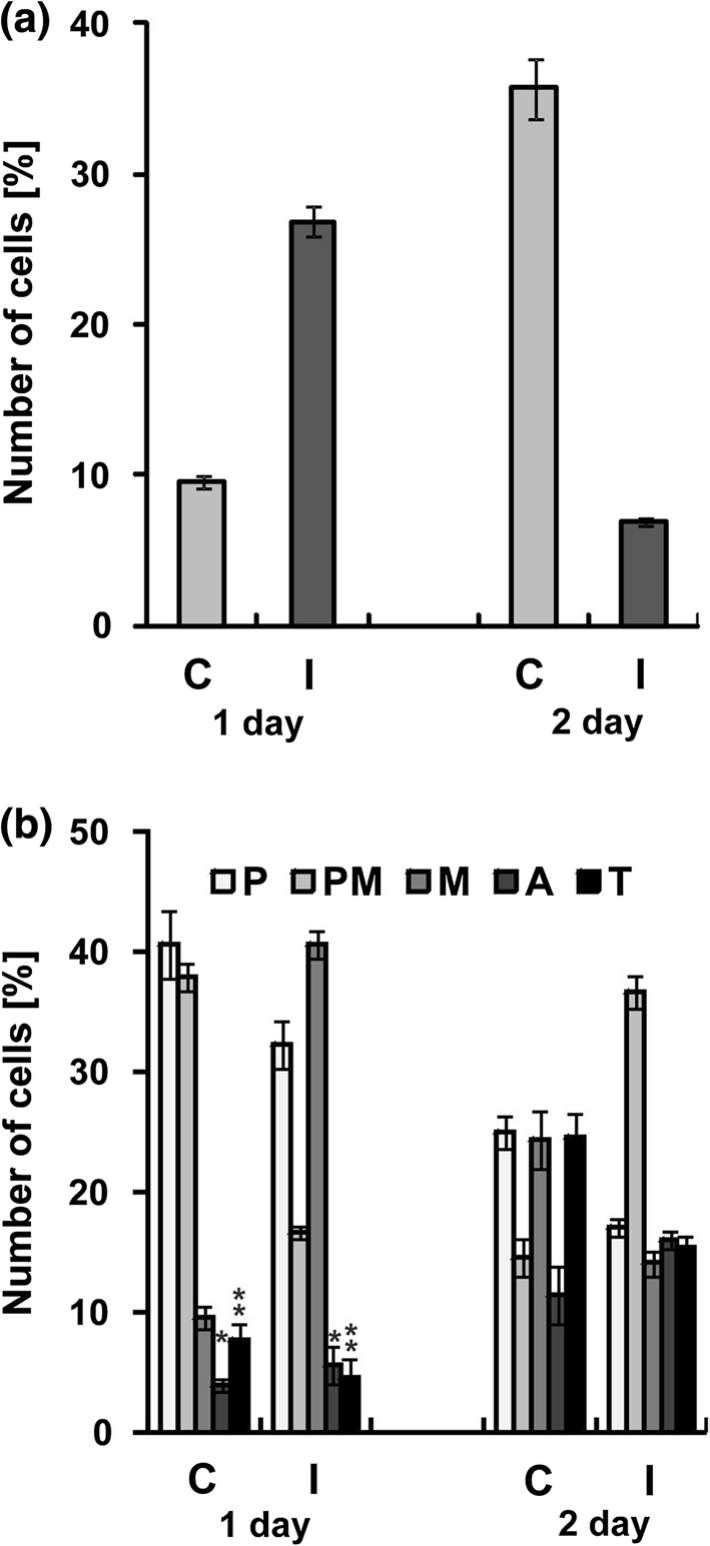
Mitotic activity in lupin root meristems after 1 and 2 days of seed germination. The seeds collected from not irrigated (control C) and irrigated (I) plants. **a** Mitotic index. **b** Phase index, prophase (P), prometaphase (PM), metaphase (M), anaphase (A), and telophase (T). Statistical significance between mean values was assessed with the two-way Anova and the post hoc unequal N HSD Tukey test (*P *< 0.01). Error bars represent standard deviation (SD). All differences between mean values presented in diagrams, except that marked with asterisk, are statistically significant

The growth of embryonic roots depends, among other, on the intensity of cell proliferation in meristems. After the first day of germination, the mitotic index (MI) in root meristems grown from the irrigated seeds was three times higher (close to 30%) than in the control (10%) (Fig. 7a). Among the mitotic cells, there were numerous metaphases, whereas in the control seeds, mainly prophases and prometaphases were observed (Fig. 7b). After the second day, the situation was reversed and a significantly higher mitotic activity was observed in the root meristems grown from the control seeds (MI = 36%) than grown from the irrigated ones (MI = 7%). The analysis of the phase indices revealed a significant percentage of cells at the all stages of division.

### Detection of hydrogen peroxide

**Fig. 8:**
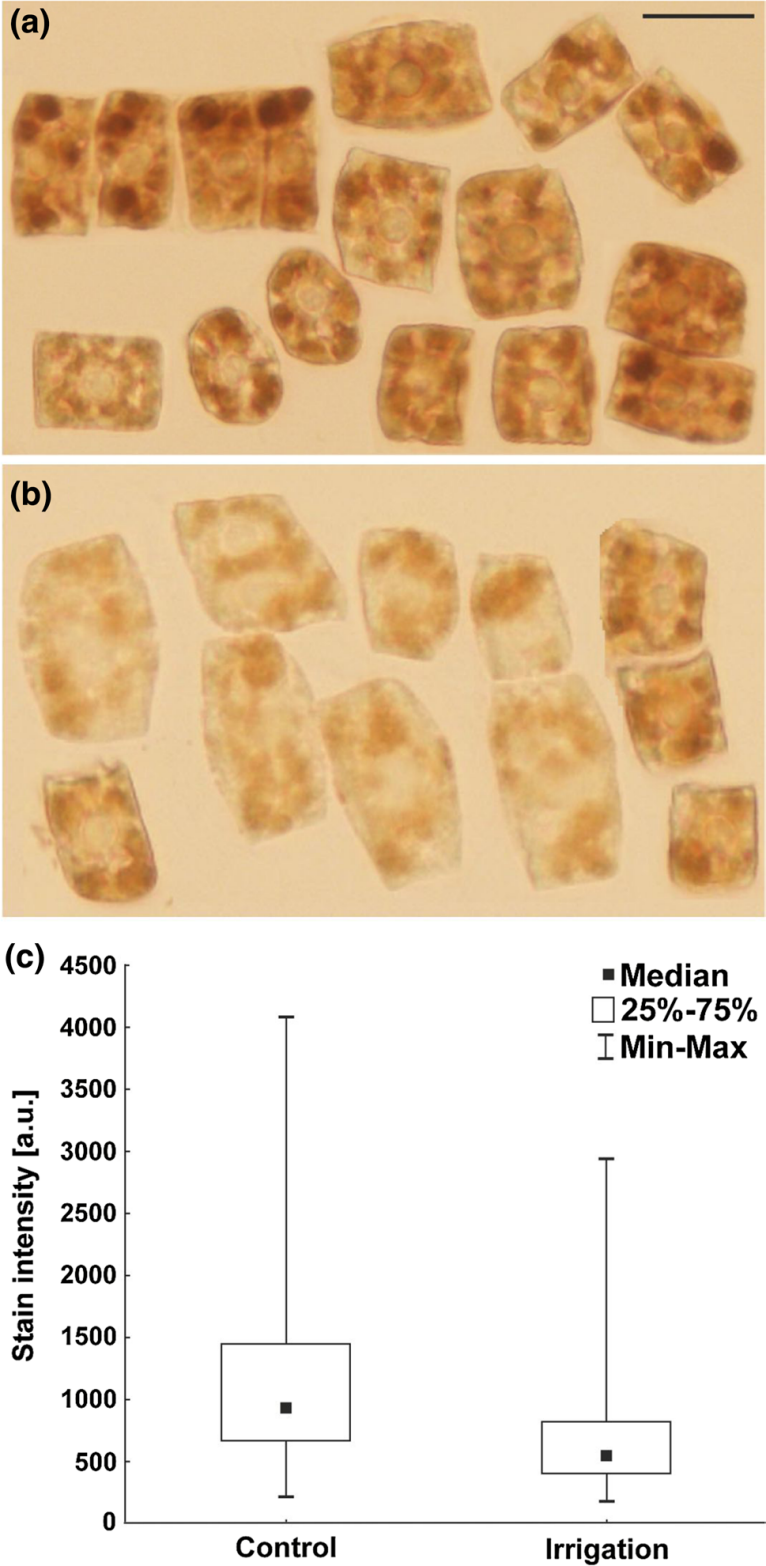
Identification of H_2_O_2_ in the form of brown DAB polymers and the level of H_2_O_2_ in the cells of embryonic roots deriving from lupin seeds. **a** Not irrigated—control plants. **b** Irrigated plants. Scale bar 20 μm. **c** Stain intensity (arbitrary units) in these cells. Statistical significance between median values was assessed with the Mann–Whitney *U* test (*P* < 0.01)

During seed germination, reactive oxygen species (e.g., H_2_O_2_) are produced in the embryo roots. Their appropriate level promotes changes, i.a. in the structure of the cell wall and elongation of cells. On the other hand, too high level of H_2_O_2_ adversely affects the cells, causing double-strand DNA breaks and damaging the structure of chromosomes. Analysis of the hydrogen peroxide content based on the DAB polymerization method revealed that its level was lower in the roots grown from irrigated seeds than in the roots of the control ones (Fig. 8).

## Discussion

Lupin plants are generally sensitive to water deficit and relatively intolerant to waterlogging, but their reactions depend on the species and plant condition (Dracup et al. 1998; Davies et al. 2000; Gresta et al. 2017). Due to changing climate and unstable weather conditions, it is a priority to find an adequate strategy aimed at maintaining and even improving legume crop yield during unfavorable water conditions. Nowadays, irrigation is one of the commonly used agrotechnical methods during water shortage. The response of lupin as well as other crops to irrigation has been studied in many different field experiments mainly focusing on plant production and yield increase, whereas mechanisms of plant responses to such conditions are still not fully understood. In the literature, there are only limited data, mostly concerning morphological changes in response of legume plants to irrigation. Our studies report a novel information related to the effects of irrigation on the narrow-leaved lupin seeds feature at the cellular level. To the best of our knowledge, the interdisciplinary research involving microscopic, cytological, biochemical, and chemical analyses of seeds collected from the irrigated and non-irrigated narrow-leaved lupin plants has not been conducted so far.

In general, irrigation, as common agrotechnical treatment, was found to increase seed yield, as long as irrigation levels do not exceed crop water requirements (Szukała and Mystek 2006; Alderfasi and Alghamdi 2010; Hill et al. 2011; Gresta et al. 2017). However, some reports indicated that irrigation may also reduce vigor and sowing value of harvested seeds (Szukała and Mystek 2006; Faligowska and Szukała 2012; Ghassemi-Golezani et al. 2012). It was demonstrated that seeds, seedlings, or mature lupin plants could differently respond to water excess; for example, in waterlogged soils, the seeds of *L. angustifolius* did not germinate and died within 4 days (Sarlistyaningsih et al. 1995). It is known that waterlogging decreased the growth of roots and root extension what can adversely affect shoot elongation, leaf expansion, and dry matter accumulation (Davies et al. 2000). The mechanisms of different plant responses, however, are not well known. Irrigation has also been found to increase infection of lupin with grey mould, leading to yield losses (Faligowska et al. 2016; Gresta et al. 2017).

Our current studies showed how irrigation of narrow-leaved lupin (*Lupinus angustifolius* cv. Baron, “sweet” variety) influenced yield and characteristics of the seeds produced by these plants. Despite the fact that irrigation of plants increased seed yield by 150%, surprisingly, this treatment contributed to the production of significantly smaller seeds—their size and weight decreased by 10% and 20%, respectively, in comparison with the seeds from non-irrigated plants (Fig. 1). The question was asked what was the reason for the inferior quality of the seed in terms of size and weight? The conducted analyses demonstrated that a low level of ploidy in the cotyledon cell nuclei was one of the factors responsible for poor seed size and weight (Fig. 2). In general, a positive correlation between cell size and ploidy level has been identified in many plants within organs, tissues, and different cell types, and has been defined as the karyoplasmic-ratio theory. This theory suggests that a driving force for cell expansion, and hence organ expansion, can be an increase in nuclear DNA content, which takes place as a result of endoreplication (Bourdon et al. 2012). Lupin seeds are filled with large cotyledons, which are reservoirs of storage substances for developing embryos and growing young seedlings. Their cells should grow quickly to create space for the next portions of synthesized storage proteins.

The environment factors, necessary or undesirable for growing plants (intensity and quality of light, temperature, hydration, salinity, heavy metals, and biotic factors: symbionts and pathogens), can have a strong impact on ploidy levels (González-Sama et al. 2006; Park et al. 2010; Chevalier et al. 2011; Scholes and Paige 2015). Drought stress, for example, in the case of wild-type *Arabidopsis*, induces the inhibition of the endoreplication process, a decrease in DNA content and cell size, consequently leading to the formation of smaller leaves (de Veylder et al. 2011). Therefore, higher levels of polyploidy are preferable for better plant expansion during drought. In the seeds from the non-irrigated lupin plants both in the basal and apical regions of cotyledons, 60% of the cells, contained mainly 8 and 16 C of the nuclear DNA. Significant reduction of the number of polyploid nuclei (to 11%) mainly visible in the apical zone of the irrigated seeds, suggested that the irrigation interfered with signaling network and could inhibit switching of the classical cell cycle to the endocycle. This mechanism is still not fully understood (Breuer et al. 2014). It is known that the ccs52 protein, found in endoreplicating cells and protein inhibitors of cyclin-dependent kinases (CKI), is of crucial importance as it inhibits cell entry into mitosis (Kondorosi et al. 2000; Breuer et al. 2014).

Spectroscopic analyses FTIR of chemical composition of lupin seeds presented in our work revealed that the structure of proteins accumulated in the seeds of the non-irrigated and irrigated plants was different (Fig. 4). Such a difference may on one hand contribute to the disturbance in the development of seeds, and on the other hand to the change of nutraceutical and taste properties of the seeds, making them unattractive for animals and humans (Hane et al. 2017), but in the case presented in this work, this remains to be determined. Protein content in lupin seeds varied between 33 and 44% in the narrow-leaved, white and yellow lupines. In general, these values are similar to the amount of protein present in soybeans (about 35%). The main proteins stored in the narrow, yellow, and white lupin seeds as well as in seeds of other legumes are globulins: γ-conglutin, β-conglutin (similar to vicilin), and α-conglutin (similar to legumin) (Lqari et al. 2004; Sujak et al. 2006; Duranti et al. 2008; Foley et al. 2015; Jimenez-Lopez et al. 2016). Although the protein content (in mg/g of fresh weight) was comparable in the control and irrigated seeds, differences in the staining intensity of electrophoretically separated protein bands (especially the most strongly stained ones, which correspond to storage proteins) may indicate a possible difference in the quantitative proportions of particular types of storage proteins (Fig. 3). Taking into account the chemical, functional, and nutritional properties of lupin proteins, its seeds can be considered as a source of high-quality protein. Therefore, preservation of the proper composition of proteins in lupin seeds during agrotechnical treatments, including irrigation, is of great importance, especially since any environmental stress could change the protein composition and structure (Battaglia and Covarrubias 2013).

Our study demonstrated that seeds produced by the irrigated plants began to germinate faster, but the growth of embryonic root was, in most cases, much weaker. Spectroscopic analyses indicated that faster germination might result from the altered seed coat cuticle composition (Fig. 4d), e.g., similar to that described by Shao et al. (2007). The plant cuticle is covered by epicuticular waxes, which play an important role in physical protection of plants against environmental conditions and insect attack. The composition of the wax mixture varies depending on plant species (Heredia 2003; Ribeiro da Luz 2006; Shao et al. 2007). Reflectance spectra of a plant surface display qualitative data related to organic constituents of its cuticle (Dubis et al. 2001). Due to the chemically changed coat of the seeds coming from the irrigated plants, the process of water absorption and seed imbibition may speed up, leading to quicker seed coat cracking and germination, similar to the situation observed in other seeds (Clua and Gimenez 2003; Clua et al. 2006).

To start the growth of the embryonic root and young seedling, the mitotic activity in meristems is required. It depends on the availability of energy and substrates for the biochemical syntheses and development of new cells. Storage substances accumulated during embryogenesis are such energy and building reserves which are used in the catabolic phase of germination. Thus, seedling germination and growth rate are directly influenced by the quality of the stored materials (Ranal and Santana 2006). To mobilize storage substances and to make them available to the embryo axis, efficient functioning of a signaling network and activation of many genes associated with germination are necessary (Gallardo et al. 2001). The main components of the signaling network are glucose, sucrose, plant hormones, and nitric oxide (Polit and Ciereszko 2009, 2012). There is a crosstalk between sugar-responsive and nitrogen-responsive pathways. Therefore, C/N balance seems to be crucial for the regulation of gene expression by carbohydrates and nitrogen, especially during germination and young seedling growth **(**for thorough analysis of the regulatory networks, see the review Osuna et al. 2015). Our elemental analysis showed lower nitrogen content in the embryo axis of the irrigated plant seeds (Fig. 6); however, other chemical contents were not significantly affected. Low N content might result from the weaker fixation of this element by bacteria. Dracup et al. (1998) indicated that waterlogging conditions can limit symbiotic nitrogen fixation by Bradyrhizobiaceae. Waterlogging may also lead to the breakdown of nodules, but once it is relieved, the plant is able to form new nodules to fix nitrogen (Davies et al. 2000). Thus, reduction of growth of seedlings might have resulted from reduced nitrogen content in the seeds of irrigated plants and an incorrect C/N ratio.

Our analysis of mitotic and phase indices indicated inefficient functioning of the signaling network regulating the cell cycle in the irrigated seeds (Fig. 7). In the meristems of germinating control seeds, the initially MI was low (after 1 day), and cell divisions took longer; that is why, the cells in prophase and prometaphase dominated. Later (after 2 days), as soon as the available nutrients appeared as a result of activation of appropriate genes, the cell cycle accelerated, MI increased and the proportions of all stages of mitosis were equal, and the roots grew faster. Immediate readiness of the irrigated plant seeds to germinate and a high MI (three times higher than in the control) observed initially in root meristems, followed by its drastic decrease (in the next days of germination) indicated that the principal control points of the cell cycle (PCP 1 in G1 phase and PCP 2 in G2 phase) received the information about direct availability of the necessary energy and building components (this may be the result of lasting catabolic reactions, which should not take place during the resting phase of seeds) and allowed to start division (Polit et al. 2003). No fast influx of new metabolites, occurring as a result of new gene expression, became an inhibitory signal for divisions in the next day (El-Maarouf-Bouteau et al. 2013). The increased prometaphase index may indicate tubulin deficiency for spindle construction or suppression of a signaling mechanism associated with the nuclear envelope breakdown (Smoyer and Jaspersen 2014).

Moreover, the lower H_2_O_2_ content in the roots germinated from irrigated seeds and their weaker growth confirmed the signal network disturbances caused by irrigation. H_2_O_2_ as a signaling molecule activates defense responses to various stresses and is one of the constitutive attributes of plant root physiology. H_2_O_2_ can also disrupt normal metabolism through oxidative damage (Moran et al. 1994; Mittler et al. 2004; Cheng and Song 2006; Cruz de Carvalho 2008). Therefore, antioxidative enzymes scavenge reactive oxygen species and protect the cells. Peroxidases (Clas III, E.C.1.11.1.7), the key enzymes in roots, can be considered as bifunctional in relation to H_2_O_2_. They catalyze the reduction of H_2_O_2_ in the peroxidative cycle converting H_2_O_2_ into water, or in the hydroxylic cycle, they can promote the formation of H_2_O_2_ (Passardi et al. 2004; Dunand et al. 2007). During seed germination, peroxidases are associated with cell-wall loosening and root elongation by generating oxygen (Liu et al. 2007; Polit et al. 2014; Szopińska 2014). Reactive oxygen species interact also with proteins which are the most sensitive molecules to oxidation. Oxidative attack of amino acyl moieties induces formation of carbonyl groups on the side chains. Carbonylation of storage proteins would help trigger their mobilization during germination (El-Maarouf-Bouteau et al. 2013). This may be the reason why seedlings from non-irrigated seeds (containing more H_2_O_2_) in our experiment grew faster.

In conclusion, our research clearly indicates that irrigation may not only affect the process of seed formation, but the pronounced effect of irrigation seems to be memorized in the seed cells and then passed on to the next generation of plants. The seedlings developing from the seeds from irrigated plants inherited the program according to which they grow. The research techniques used in our research demonstrated that irrigation triggered long-term effects and the seed response was a multilevel one. It is, therefore, extremely important to know the mechanisms that control the response of plants to irrigation. Apart from the cognitive aspect, the acquired knowledge will also bring economic benefits allowing to eliminate inadequate agronomic practices and to avoid losses due to the storage of faulty crop.

### *Author contribution statement*

GS conceived and designed a field research. IC and JP conceived and designed a laboratory research. AF and ŁS conducted a field experiment and provided seeds. JL, AD, AB, AŻ, MH, KK, and JP conducted laboratory experiments. KK conducted statistical analysis. IC, KK, and JP analyzed data. KK and JP wrote the manuscript. JM conducted a language correction. All authors read and approved the manuscript.

## Electronic supplementary material

Below is the link to the electronic supplementary material.

Supplementary material 1 (PDF 156 kb)

Supplementary material 2 (PDF 158 kb)

Supplementary material 3 (PDF 156 kb)

## Abbreviations

FTIR: Fourier transform infrared spectroscopy
SEM: Scanning electron microscope
