# Correction to: An International Journal of Plant Biology

**DOI:** 10.1007/s00425-019-03165-8

**Published:** 2019-04-17

## Correction to: Planta 10.1007/s00425-019-03091-9, 10.1007/s00425-019-03117-2

The articles listed above were initially published with incorrect copyright information.

Upon publication of this Correction, the copyright of these articles changed to “The Author(s)”.

The original articles have been corrected.

