# Supplementary material for: Irrigation affects characteristics of narrow-leaved lupin (Lupinus angustifolius L.) seeds
Source: Planta. 2019 Jan 25;249(6):1731–46. doi: 10.1007/s00425-019-03091-9 (PMC12125071; doi:10.1007/s00425-019-03091-9)
Supplement: Supplementary file 1 — Supplementary material 1 (PDF 156 kb) [file 425_2019_3091_MOESM1_ESM.pdf]

article title – Irrigation affected chemical composition and quality of seeds of narrow-leaved lupin (*Lupinus angustifolius* L.)

journal name – Planta

author names – Konrad Winnicki[1], Iwona Ciereszko[2], Joanna Leśniewska[2] Alina T. Dubis[3], Anna Basa[3], Aneta Żabka[1], Marcin Hołota[1], Łukasz Sobiech[4], Agnieszka Faligowska[4], Grzegorz Skrzypczak[4], Janusz Maszewski[1], Justyna T. Polit[1]*,

affiliation –

1 Department of Cytophysiology, Faculty of Biology and Environmental Protection, University of Łódź, Pomorska 141/143, 90-236 Łódź, Poland

2 Institute of Biology, Faculty of Biology and Chemistry, University of Bialystok, Ciołkowskiego1J, 15-245 Bialystok, Poland

3 Institute of Chemistry, Faculty of Biology and Chemistry, University of Bialystok, Ciołkowskiego1K, 15-245 Bialystok, Poland

4 Agronomy Department, Poznań University of Life Sciences, Dojazd 11, 60-632 Poznań, Poland

e-mail address of the corresponding author - justyna.polit@biol.uni.lodz.pl

ESM1

Suppl. Table S1

Date, dose of water and time of irrigation during vegetation period of narrow-leaved lupin (*Lupinus angustifolius* cultivar Baron).

Date	Dose of water (mm)	Time of irrigation (h)
May 25	30	6
June 1	30	6
June 6	35	7
June 13	35	7
June 29	35	7
Sum	165	33
