# Supplementary material for: Irrigation affects characteristics of narrow-leaved lupin (Lupinus angustifolius L.) seeds
Source: Planta. 2019 Jan 25;249(6):1731–46. doi: 10.1007/s00425-019-03091-9 (PMC12125071; doi:10.1007/s00425-019-03091-9)
Supplement: Supplementary file 3 — Supplementary material 3 (PDF 156 kb) [file 425_2019_3091_MOESM3_ESM.pdf]

article title – Irrigation affected chemical composition and quality of seeds of narrow-leaved lupin (*Lupinus angustifolius* L.)

journal name – Planta

author names – Konrad Winnicki[1], Iwona Ciereszko[2], Joanna Leśniewska[2] Alina T. Dubis[3], Anna Basa[3], Aneta Żabka[1], Marcin Hołota[1], Łukasz Sobiech[4], Agnieszka Faligowska[4], Grzegorz Skrzypczak[4], Janusz Maszewski[1], Justyna T. Polit[1]*,

affiliation –

1 Department of Cytophysiology, Faculty of Biology and Environmental Protection, University of Łódź, Pomorska 141/143, 90-236 Łódź, Poland

2 Institute of Biology, Faculty of Biology and Chemistry, University of Bialystok, Ciołkowskiego1J, 15-245 Bialystok, Poland

3 Institute of Chemistry, Faculty of Biology and Chemistry, University of Bialystok, Ciołkowskiego1K, 15-245 Bialystok, Poland

4 Agronomy Department, Poznań University of Life Sciences, Dojazd 11, 60-632 Poznań, Poland

e-mail address of the corresponding author - justyna.polit@biol.uni.lodz.pl

ESM3

Suppl. Table S3

Seed yield of narrow-leaved lupin [g/plant] harvested from the main stems, branches and whole control (not irrigated) and irrigated plants. Statistical significance between mean values was assessed with the one-way Anova and the post-hoc LSD (Least Significant Difference) test.

	Main Stem	Branches	Plant
Control	0.8	1.58	2.38
Irrigation	2.17	3.75	5.92
LSD (0.05)	0.689	1.979	2.437
